# Correlation of carotid blood flow and corrected carotid flow time with invasive cardiac output measurements

**DOI:** 10.1186/s13089-017-0065-0

**Published:** 2017-04-20

**Authors:** Irene W. Y. Ma, Joshua D. Caplin, Aftab Azad, Christina Wilson, Michael A. Fifer, Aranya Bagchi, Andrew S. Liteplo, Vicki E. Noble

**Affiliations:** 1 Division of Emergency Ultrasound, Department of Emergency Medicine, Massachusetts General Hospital, 326 Cambridge Street, Suite 410, Boston, MA 02114 USA; 2 Division of General Internal Medicine, Department of Medicine, University of Calgary, 3330 Hospital Dr NW, Calgary, AB T2N 4N1 Canada; 3 Cardiology Division, Department of Medicine, Massachusetts General Hospital, 15 Parkman Street #800, Boston, MA 02114 USA; 4 Department of Emergency Medicine, Hamad Medical Corporation, PO Box 3050, Doha, Qatar; 5 Department of Anaesthesia, Massachusetts General Hospital, 55 Fruit Street, Boston, MA 02114 USA; 6 Department of Emergency Medicine, University Hospitals, Cleveland Medical Center, Case Western Reserve University, 11100 Euclid Ave, Cleveland, OH 44106 USA

**Keywords:** Cardiac output, Carotid ultrasound, Carotid flow time, Carotid blood flow

## Abstract

**Background:**

Non-invasive measures that can accurately estimate cardiac output may help identify volume-responsive patients. This study seeks to compare two non-invasive measures (corrected carotid flow time and carotid blood flow) and their correlations with invasive reference measurements of cardiac output. Consenting adult patients (*n* = 51) at Massachusetts General Hospital cardiac catheterization laboratory undergoing right heart catheterization between February and April 2016 were included. Carotid ultrasound images were obtained concurrently with cardiac output measurements, obtained by the thermodilution method in the absence of severe tricuspid regurgitation and by the Fick oxygen method otherwise. Corrected carotid flow time was calculated as systole time/√cycle time. Carotid blood flow was calculated as *π* × (carotid diameter)^2^/4 × velocity time integral × heart rate. Measurements were obtained using a single carotid waveform and an average of three carotid waveforms for both measures.

**Results:**

Single waveform measurements of corrected flow time did not correlate with cardiac output (*ρ* = 0.25, 95% CI −0.03 to 0.49, *p* = 0.08), but an average of three waveforms correlated significantly, although weakly (*ρ* = 0.29, 95% CI 0.02–0.53, *p* = 0.046). Carotid blood flow measurements correlated moderately with cardiac output regardless of if single waveform or an average of three waveforms were used: *ρ* = 0.44, 95% CI 0.18–0.63, *p* = 0.004, and *ρ* = 0.41, 95% CI 0.16–0.62, *p* = 0.004, respectively.

**Conclusions:**

Carotid blood flow may be a better marker of cardiac output and less subject to measurements issues than corrected carotid flow time.

**Electronic supplementary material:**

The online version of this article (doi:10.1186/s13089-017-0065-0) contains supplementary material, which is available to authorized users.

## Background

To be able to identify patients who are fluid responsive is important in the management of those who are acutely ill. At present, there is no non-invasive method that can reliably and accurately identify fluid responsiveness. As such, in patients with undifferentiated shock, treatment often involves empiric fluid administration, in the hopes that volume expansion will increase preload, which will then serve to increase cardiac output (CO). However, for patients on the flat portion of the Starling curve, aggressive fluid administration results in no appreciable increase in CO and may be detrimental [1–3]. Thus, the ability to identify where each patient is on his/her Starling curve can help identify patients who would benefit from additional fluid (fluid responsive) and those who would not (fluid unresponsive). Unfortunately, traditional measures of preload such as central venous pressure have not consistently been shown to be helpful in identifying volume responsiveness [4, 5]. The use of pulse pressure variation shows promise in ventilated patients, but requires the insertion of an arterial line [5]. As such, there remains a pressing need to be able to identify fluid responsiveness non-invasively at the bedside.

Fluid responsiveness is typically defined as an increase in CO by 10–15% in response to fluid administration [6]. To avoid excess fluid administration, an increase of CO in response to maneuvers such as the passive leg raise (PLR) is considered indicative of fluid responsiveness [7]. PLR is typically performed with the patient either in the supine or semirecumbent position, followed by repeat measurements with the patient’s legs passively raised at 30–45° [7]. This maneuver is considered to result in an auto-bolus of fluid of approximately 300 mL in volume [8]. The benefit of the PLR is that its hemodynamic effects are rapidly reversible, since no fluid administration actually takes place.

Cardiac output, however, has been challenging to measure at the bedside. The traditional reference standard for measuring CO requires the insertion of a pulmonary artery catheter [5, 9] which is invasive, associated with a risk for serious complications [10], and its use may offer no clinical benefits [11–15]. Newer non-invasive devices using bioreactance parameters have mixed evidence in their accuracy and reliability [16–20], and require a dedicated machine that may not be readily available. Lastly, estimations of CO using echocardiography have been suggested as a bedside measure. However, its use may not be feasible due to high training requirements [21].

In the quest to identify feasible, non-invasive, and reproducible bedside estimates of CO, carotid Doppler imaging shows promise. In particular, two carotid measurements have emerged as candidate markers of CO: corrected carotid flow time (CFT) and carotid blood flow (CBF). CFT is the carotid systole time, with heart rate correction applied. This measure is easy to perform and may correlate with intravascular volume [22]. CBF is the integral of blood volume that is ejected through the carotid artery with each cardiac cycle. This measure has been shown to be feasible to perform at the bedside [23, 24].

Studies to date have shown that corrected CFT increases in response to fluid administration or consumption [25, 26], and decreases in response to volume removal in dialysis [22] and blood donation [27]. However, none of these studies correlated corrected CFT with CO. CBF has been less extensively studied. However, in one study of 34 patients, a change in CBF in response to PLR was found to correlate significantly with a change in stroke volume index, measured by bioreactance [28].

Despite these promising studies, neither measure has been correlated directly with a commonly used invasive reference standard for measuring CO via the pulmonary artery catheter. As such, this study seeks to compare corrected CFT and CBF with invasive measures of CO.

## Methods

All adult patients at Massachusetts General Hospital cardiac catheterization laboratory undergoing right heart catheterization between February and April 2016 were invited to participate. Non-consenting patients, patients on mechanical ventilation, and those unable to tolerate a passive leg raise (PLR) maneuver were excluded (e.g., unable to lie supine or had pain with PLR).

Carotid ultrasound images were obtained concurrently with CO measures with the patient in the supine position. A PLR was then performed by elevating the legs using a standardized 30° foam wedge (RayShield^®^ AADCO Medical Inc., Vermont). Repeat carotid ultrasound images and concurrent CO measurements were then obtained within 1 min after PLR, as maximal blood flow changes were felt to be observed within 1 min [8, 29].

Carotid ultrasound images were obtained by an emergency ultrasound fellow (I.M.). Images were obtained using a linear-array (15–4 MHz) transducer on a bedside ultrasound system (uSmart^®^ Terason 3200T, Burlington, MA). The carotid vascular preset was used (pulse repetition frequency 5.0 kHz, wall filter 75 Hz). The common carotid artery was scanned in transverse and longitudinal planes. Spectral Doppler tracings were then obtained by placing a 0.5 mm sample gate through the center of vessel, within 2–3 cm proximal to the carotid bulb in the longitudinal plane, in accordance to standard guidelines [30]. The angle correction cursor was placed parallel to the direction of blood flow. Images with insonation angles >60° were excluded because of resultant inaccuracies of flow and velocity measurements at such angles [31].

### Carotid measurements

**Fig. 1:**
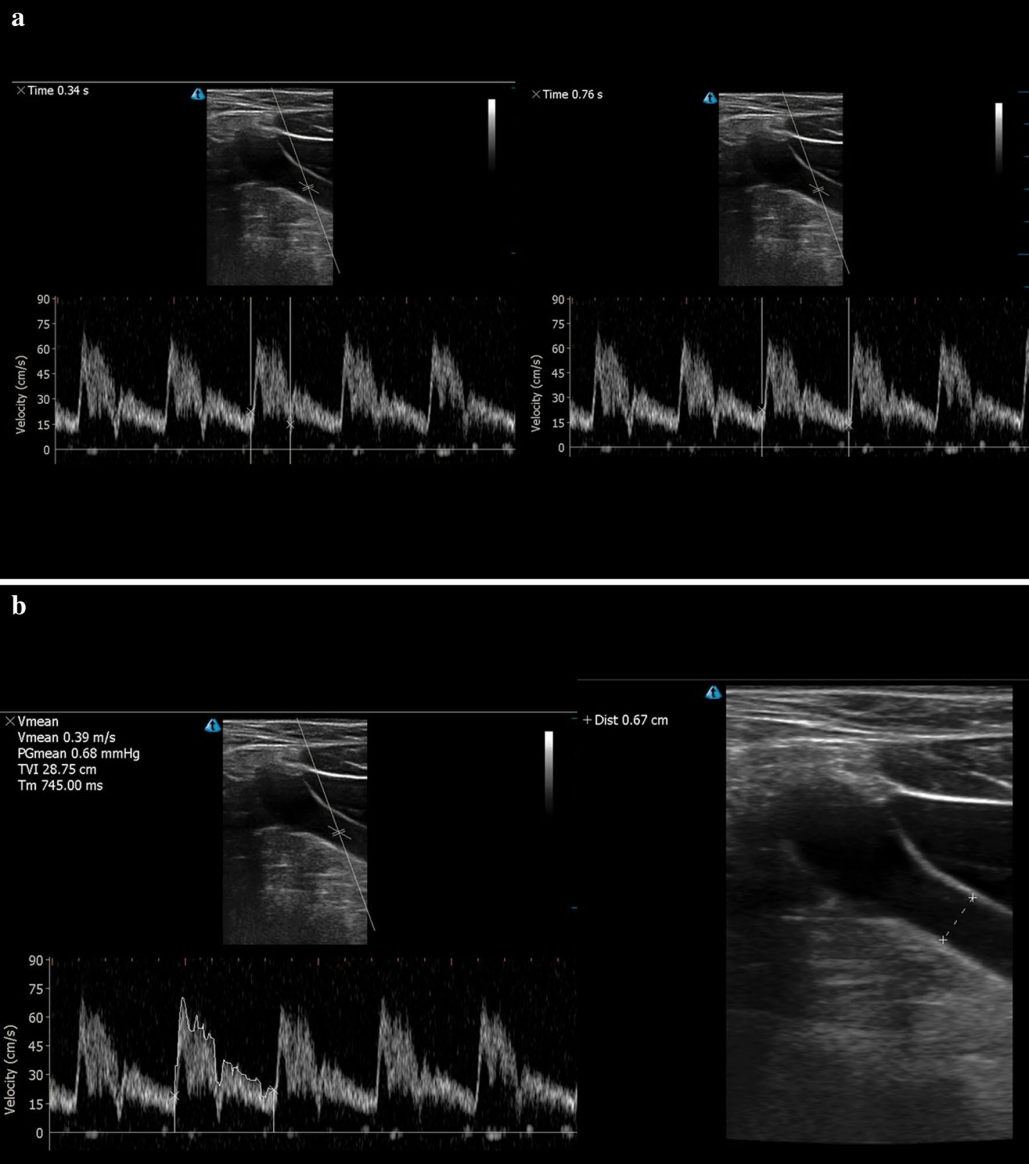
**a** Carotid systole time, as measured from the start of the systolic upstroke to the start of the dicrotic notch (*left*). Carotid cycle time, as measured from start of one systolic upstroke to the next (*right*). **b** Velocity time integral tracing of the spectral Doppler signal (*left*). Carotid diameter (*right*)

Corrected CFT was calculated as systole time/√cycle time [27]. Systole time was measured from the start of systolic upstroke to the start of the dicrotic notch, while cycle time was the duration of the cycle (Fig. 1a).

CBF was calculated as

$${\text{blood flow}} \; = \; \pi \; \times \; \left( {\text{carotid diameter}} \right)^{ 2} / 4 \; \times \;{\text{VTI}}\; \times \; {\text{heart rate}} ,$$

where VTI indicates velocity time integral [28]. VTI of the Doppler signal was measured using manual tracings (Fig. 1b). Intimal-to-intimal carotid diameter was measured at the level of the sample gate (Fig. 1b).

All carotid measurements (systole time, cycle time, VTI, diameter) were measured on a single waveform by one investigator (I.M.) in order to calculate corrected CFT and CBF. Average corrected CFT and average CBF were then calculated using a mean of three waveforms. To establish interrater reliability on obtaining measurements, all measurements on obtained images were then repeated by a second independent investigator (C.W. or A.A., both with similar training to I.M.), who was blinded to all CO and carotid measurements. Results from the second investigator were used only to provide interrater reliability estimates.

Image acceptability was rated independently by two trained raters (C.W., A.A.), using five domains (internal reliability acceptable, Cronbach’s alpha = 0.84): correction angle parallel to vessel, sample gate in the center of the vessel, sufficient gain, vessel was non-oblique, and measurements made within 2–3 cm proximal to carotid bulb (see Additional file 1). All domains were rated using a 5-point Likert scale, where 1 = poor quality and 5 = excellent quality, with overall image quality rated as a summary measure, based on global expert opinion.

Cardiac output measurements were obtained by thermodilution method in the absence of severe tricuspid regurgitation, by an injection of 10 mL of sterile 0.9% saline injection into the proximal lumen of the pulmonary artery catheter and subsequent detection of the temperature change at the distal thermistor [32]. Fick oxygen method was used in the presence of severe tricuspid regurgitation [33]. The Fick method involved simultaneous arterial and mixed venous blood sample measurement, and cardiac output was determined based upon the ratio between estimated oxygen consumption and arteriovenous oxygen gradient [34]. Post hoc analyses of correlation measures were performed in subgroups of patients in and not in atrial fibrillation at the time of the procedure.

This study was approved by the Partners Human Research Committee Institution Review Board.

### Statistical analysis

On the basis of Marik et al.’s prior study on estimates of stroke volume indices correlations with carotid blood flow [28], we estimated that 26 patients would have 90% power to detect a correlation of 0.59 (*α* = 0.05). However, as interim analyses revealed a lower than 0.59 correlation in our study sample. A repeat sample size calculation suggested that an estimated 48 patients would be needed to detect a correlation of 0.45 (*α* = 0.05, power = 0.9).

Pre- and post-PLR measurements were compared using paired *t* tests and Wilcoxon signed-rank tests, as appropriate. Correlations between CO and carotid parameters were made using Spearman’s rho, a non-parametric correlation coefficient. Confidence intervals around Spearman’s rho were based on Fisher’s r to z transformation [35]. Correlation coefficients of 0.10–0.29 were considered weak, 0.30–0.49 as moderate, and 0.50–1.00 as strong [36]. All *p* values were adjusted for multiple comparisons using the Benjamini–Hochberg procedure [37], which allows the false discovery rate to be controlled at the 0.05 level.

All analyses were performed using SAS 9.4 (SAS Institute Inc. Cary, NC) and SPSS version 24 (IBM Corp. Armonk, NY).

## Results

**Fig. 2:**
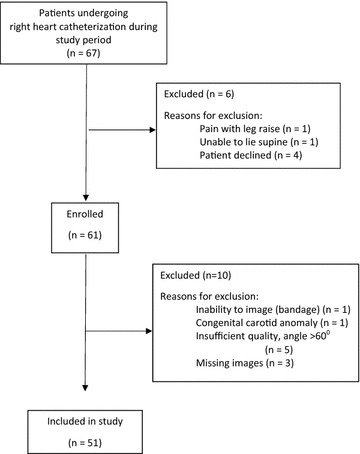
Flow chart of number of patients enrolled in the study and included in the analyses

Of the 67 eligible patients, 51 patients (76%) were included in the final analysis (Fig. 2; Table 1). The majority of patients had cardiac output measurements done using thermodilution (*n* = 44; 86%). Five patients were excluded because of angles of insonation of >60°. Of the included 51 patients, median angle of insonation was 60°, with a range of 38–60°. Overall image quality of the included patients was rated as above-average quality [mean score 4.1 ± standard deviation (SD) 0.6] based on global expert opinion. As there were no differences in CO, corrected CFT, or CBF between pre- and post-PLR maneuver (Table 2), only baseline measures (i.e., pre-PLR) will be further discussed. Interrater reliability for corrected CFT and CBF measurements on obtained images was excellent [intraclass correlation coefficient (ICC) 0.90, 95% CI 0.82–0.94; ICC 0.96, 95% CI 0.58–0.99, respectively].

**Table 1:** Baseline characteristics of 51 patients included in the study

Baseline characteristics	Number (%)
Mean age in years ± standard deviation	59.6 ± 16.3
Gender	
Male	39 (76)
Female	12 (24)
Body mass index (kg/m^2^) ± standard deviation	26.3 ± 5.9
History of	
Diabetes mellitus	13 (25)
Hypertension	26 (51)
Dyslipidemia	22 (43)
Coronary artery disease	17 (33)
Prior angioplasty and/stent placement	7 (14)
Prior coronary bypass surgery	4 (8)
Stroke or transient ischemic attacks	11 (22)
Atrial fibrillation	13 (25)
Moderate or severe aortic insufficiency	1 (2)
Moderate or severe aortic stenosis	5 (10)
Indication for right heart catheterization	
Diagnostic right heart catheterization only	24 (47)
Cardiac biopsy	27 (53)
Cardiac output measurement method	
Thermodilution	44 (86)
Fick oxygen method	7 (14)
Vascular access	
Internal jugular	40 (78)
Forearm (cephalic or basilic)	5 (10)
Femoral	6 (12)

**Table 2:** Baseline mean cardiac output and median carotid ultrasound parameters (corrected flow time and blood flow) pre- and post-passive leg raise

Parameter	Pre-passive leg raise median (interquartile range)	Post-passive leg raise median (interquartile range)	*p* value
Mean cardiac output ± standard deviation (SD) (L/min)	5.15 ± 1.58	5.19 ± 1.58	0.69
Corrected flow time (single waveform in milliseconds)	331.7 (308.6–357.8)	344.2 (321.6–360.6)	0.06
Corrected flow time (average of three waveforms in milliseconds)	335.2 (311.0–359.3)	339.6 (322.1–368.9)	0.13
Blood flow (single waveform in mL/min)	576.3 (389.5–806.9)	551.77 (441.5–763.5)	0.45
Blood flow (average of three waveforms in mL/min)	555.4 (422.1–766.6)	558.3 (452.7–741.6)	0.45

### Corrected carotid flow time (CFT)

Single waveform measurements of corrected CFT did not correlate with CO measurements [*ρ* = 0.25, 95% confidence interval (CI) −0.03 to 0.49, adjusted *p* = 0.08]. Corrected CFT measurements using three waveforms correlated significantly, but weakly, with CO (*ρ* = 0.29, 95% CI 0.02–0.53, adjusted *p* = 0.046).

### Carotid blood flow (CBF)

Single waveform CBF correlated moderately and significantly with CO [*ρ* = 0.44, 95% CI 0.18–0.63, adjusted *p* = 0.004). CBF measurements using three waveforms also moderately and significantly correlated with CO (*ρ* = 0.41, 95% CI 0.16–0.62, adjusted *p* = 0.004).

### Subgroup analysis

In a subgroup of patients not in atrial fibrillation at the time of the procedure (*n* = 45), single waveform measurements of corrected CFT did not correlate with CO (*ρ* = 0.26, 95% CI −0.04 to 0.51, adjusted *p* = 0.17). For patients in atrial fibrillation at the time of the procedure (*n* = 6), single waveform corrected CFT measurements did not correlate with CO (*ρ* = 0.09, 95% CI −0.78 to 0.84, adjusted *p* = 0.88). In patients not in atrial fibrillation (*n* = 45), corrected CFT measurements using three waveforms correlated moderately and significantly with CO (*ρ* = 0.33, 95% CI 0.04–0.56, adjusted *p* = 0.03). In patients in atrial fibrillation (*n* = 6), corrected CFT measurements using three waveforms did not correlate with CO (*ρ* = −0.14, 95% CI −0.86 to 0.76, adjusted *p* = 0.92).

Single waveform measurements of CBF correlated moderately and significantly with CO in patients not in atrial fibrillation at the time of the procedure (*ρ* = 0.42, 95% CI 0.15–0.64, adjusted *p* = 0.03). For the six patients in atrial fibrillation, single waveform measurements did not correlate with CO (*ρ* = 0.66, 95% CI −0.33 to 0.96, adjusted *p* = 0.28). In patients not in atrial fibrillation (*n* = 45), CBF measurements using three waveforms correlated moderately and significantly with CO (*ρ* = 0.40, 95% CI 0.12–0.62, adjusted *p* = 0.02). For the six patients in atrial fibrillation at the time of the procedure, CBF measurements using three waveforms did not correlate with CO (*ρ* = 0.60, 95% CI −0.41 to 0.95, adjusted *p* = 0.31).

### Self-report feasibility

Overall, reported ease of carotid diameter measurement was high (mean score 4.1 ± SD 0.8, where 1 = very difficult and 5 = very easy). Reported ease of measuring VTI tracings was moderate (mean score 3.5 ± SD 0.8). Ease of systole time was moderate (3.7 ± SD 0.8) and high for cycle time (4.2 ± SD 0.7).

## Discussion

In this study of 51 adult patients undergoing invasive right heart catheterization and simultaneous carotid ultrasound, CBF correlated moderately and significantly with CO. This relationship was present whether a single waveform or an average of three waveforms was used. Corrected CFT, on the other hand, when measured using only a single waveform, as per the method used by prior studies [22, 25–27], demonstrated no correlation with CO. Only by measuring three waveforms was corrected carotid flow time’s correlation with CO significant. However, the strength of this relationship was weak. Altogether, our findings suggest CBF may be a better surrogate marker for CO and that CBF measurements may be less subject to measurements issues than corrected CFT.

The ability to estimate CO in an accurate, reliable, and feasible manner is an important part of determining patients’ volume responsiveness. Carotid ultrasound is a promising tool for two reasons. First, unlike the traditional reference standard of CO measurement from a pulmonary artery catheter [5, 9], carotid ultrasound imaging is non-invasive and readily available in many centers. Second, as evidenced by reported ease of measurements in our study, and confirmed by prior studies [23, 24], carotid ultrasound is easy to perform. This is in contrast to echocardiographic measures of CO [21], which requires more extensive training [38], as its accuracy is dependent on scan techniques and patient factors such as obesity and availability of sonographic windows [39].

In deciding whether to use corrected CFT or CBF as an estimate of CO, one should consider the following factors: (1) strength of the correlation with reference standard, (2) contextual factors that may limit its accuracy, (3) reliability, (4) feasibility, and (5) sensitivity in detecting CO changes. We argue that CBF is superior for the first two factors, and that both CBF and corrected CFT demonstrated high interrater reliability and feasibility.

To our knowledge, ours is the first study to compare both carotid ultrasound measures with invasive measures of CO. Our study demonstrates that CBF correlates stronger than corrected CFT with CO and is less sensitive to measurements errors. We hypothesize that two sources of errors may be contributing to measurement errors in corrected CFT: cardiac arrhythmia and underlying cardiac conditions.

With respect to the cardiac arrhythmia hypothesis, we noted that corrected CFT measurements based on averaging three waveforms yielded a higher and significant correlation, compared to measurements based only on one waveform. The potential of respiratory contribution to the variations in cycle time and systole time (sinus arrhythmia) may contribute to this finding. The hypothesis regarding the contribution of arrhythmias to inaccurate measurements was further supported by findings from our subgroup analysis, whereby the correlation of corrected CFT with CO became significant in a subgroup of patients not in atrial fibrillation at the time of the procedure, and only if an average of three waveforms were used. Correlations were lower in general for patients in atrial fibrillation compared with those not in atrial fibrillation, whether a single waveform or three waveforms were used. Indeed, prior studies evaluating corrected carotid flow time excluded patients with atrial fibrillation [22, 25–27]. In our subgroup analysis of patients in atrial fibrillation, we were unable to detect significant correlations with cardiac output measurement regardless of whether CBF or corrected CFT was used, and regardless of whether a single waveform or an average of three waveforms was used. However, our small sample size (*n* = 6) is likely too small to demonstrate a significant correlation. Future studies should evaluate the use of these parameters in patients in atrial fibrillation. Until such studies are performed, it may be prudent to continue to exclude patients with significant cardiac arrhythmia when attempting to use carotid measurements as surrogates for CO.

With respect to the cardiac condition hypothesis, it is noteworthy that measurements of systole time require the identification of the start of the dicrotic notch. Valvular diseases are known to affect carotid tracings, which may render the dicrotic notch more difficult to appreciate [40–42]. Indeed, in our study, self-report ease of measuring cycle time was higher than for systole time, which may be a reflection of the difficulty in identifying the dicrotic notch in certain cases. Carotid blood flow, on the other hand, utilizes more clinical parameters (diameter, VTI, and heart rate) that may be less subject to the changes of any single parameter and may therefore more accurately estimate for the volumetric flow of blood through the carotid. For example, adaptation to a lower VTI may be accommodated by a larger carotid diameter and/or a faster heart rate. Therefore, accounting for these additional parameters may result in an overall more accurate estimate.

There are a number of limitations in our study. First, this is a single-centered study. Second, we are able to report only interrater reliability for image interpretation and not for image acquisition (a single sonographer performed all the scanning). Image acquisition by untrained sonographers may potentially result in unacceptable interrater reliability. Future studies should examine image acquisition interrater reliability and the role of training. Third, as our patients were largely euvolemic, PLR maneuvers resulted in no demonstrable changes in any of our measured parameters. Therefore, we cannot comment on the sensitivity of carotid measurements to changes imposed by dynamic maneuvers. Further, our use of a 30° foam wedge rather than the more commonly used 45° leg lift [7] may have further limited our ability to induce significant changes pre- and post-PLR. In addition, although it has been argued that the maximal blood flow changes occur within one minute [8, 29], the majority of studies evaluating PLR use a longer time frame [7]. The optimal timing remains unclear. Fourth, our subgroup analysis of patients in and not in atrial fibrillation was performed in a post hoc manner. Results from these analyses should be considered as hypothesis generating only. Fifth, our carotid VTI measurements were performed manually. Therefore, measurement errors may be present. However, measurement errors would be expected to dilute correlation coefficients towards the null [43]. Because many machines are equipped with automatic VTI tracings, correlation coefficients for CBF measured in an automated manner may yield higher correlations than those reported in our study. Sixth, we do not have information on the history of carotid disease. Therefore, we are unable to determine the impact of carotid disease on the accuracy of our carotid ultrasound measurements. Last but not least, the majority of our patients underwent the thermodilution method for cardiac output estimation. While its use is commonly accepted as the practical gold standard, its use is not without limitations, especially in the presence of significant tricuspid regurgitation, intracardiac shunts, or concurrent intravenous infusions [9, 44, 45].

## Conclusions

Our study demonstrates that compared to corrected CFT, CBF demonstrated stronger and more consistent correlation with CO. Its use for assessing volume responsiveness should be further evaluated.

## Additional file

**Additional file 1.** Carotid artery doppler quality assessment tool (CADQAD).

## Abbreviations

CBF: carotid blood flow
CFT: carotid flow time
CO: cardiac output
CI: confidence interval
ICC: intraclass correlation coefficient
PLR: passive leg raise
SD: standard deviation
VTI: velocity time integral
