# Supplementary material for: Correlation of carotid blood flow and corrected carotid flow time with invasive cardiac output measurements
Source: Crit Ultrasound J. 2017 Apr 20;9:10. doi: 10.1186/s13089-017-0065-0 (PMC5398973; doi:10.1186/s13089-017-0065-0)
Supplement: Supplementary file 1 — Additional file 1. Carotid artery doppler quality assessment tool (CADQAD). [file 13089_2017_65_MOESM1_ESM.docx]

| Please rate the following areas | Poor Quality | Borderline Quality and **Insufficient** | Borderline Quality but **Sufficient** | Above average Quality | Excellent Quality | Unable to assess |
| --- | --- | --- | --- | --- | --- | --- |
| Correction angle  parallel to vessel |  |  |  |  |  |  |
| SV gate in center  of vessel |  |  |  |  |  |  |
| Sufficient gain |  |  |  |  |  |  |
| Non-oblique |  |  |  |  |  |  |
| Within 2-3 cm  proximal to bulb |  |  |  |  |  |  |
| **OVERALL QUALITY** |  |  |  |  |  |  |

**Additional File 1. Carotid Artery Doppler Quality Assessment Tool (CADQAD)**

**ID #: _______________ Rater:______________**

**Comments:**
